# The Tape of Life: A Musical Activity Engaging the Public with Aging Research

**DOI:** 10.1093/geroni/igaf122.4376

**Published:** 2025-12-31

**Authors:** Jessica Foley

**Affiliations:** Tufts University, Medford, Massachusetts, United States

## Abstract

Effective science communication can come in many different forms. In particular, the use of artistic approaches can help to make complex concepts more appealing and accessible to new audiences. Concerns about the globally aging population, along with growing media coverage of the anti-aging industry, make public engagement with aging research of particular importance. Considering the current emphasis on technology-enhanced learning, and the proliferation of accessible tools for music production, we devised an interactive music-based science engagement activity designed to introduce new audiences to the diversity of animal aging, and to explore attitudes to longevity research. Participants recorded a snippet of themselves singing into our “lifespan-emulator” (a musical synthesizer ) and listened as their “tape of life” sped up, decayed, and echoed to mirror the lifespan, aging rate, and reproductive curve of several different animal species. We then used this musical activity as a launching point for more in-depth discussions of the biology of aging, and to explore public perception of work aiming to extend the human lifespan. We presented this at five UK-based science festivals, introducing several hundred participants from diverse demographic backgrounds to aging research. Participants’ attitudes were evaluated using non-interventionist methods such as poll questions and drawing activities. We found that broadly, our audiences were not interested in substantial extensions to human longevity, with the average “ideal” human lifespan approximately that of the current maximum. Our work demonstrates the efficacy of creative approaches to science communication, and the value of public engagement as a tool to inform research.

